# Validity of calendar day-based definitions for community-onset bloodstream infections

**DOI:** 10.1186/s13104-015-1051-x

**Published:** 2015-04-02

**Authors:** Kevin B Laupland, Daniel B Gregson, Deirdre L Church

**Affiliations:** Department of Medicine, Royal Inland Hospital, 311 Columbia Street, Kamloops, BC V2C 2T1 Canada; Department of Medicine, University of Calgary, Calgary, AB Canada; Department of Pathology and Laboratory Medicine, University of Calgary, Calgary, AB Canada; Division of Microbiology, Calgary Laboratory Services, #9, 3535 Research Road NW, Calgary, AB T2A 2K8 Canada

**Keywords:** Nosocomial, Bacteremia, Community-acquired, Incidence, Epidemiology

## Abstract

**Background:**

Community-onset (CO) bloodstream infections (BSI) are those BSI where the blood culture is drawn <48 hours from hospital admission. However, exact times of culture draw or hospital admission are not always available. We evaluated the validity of using 2- or 3- calendar day based definitions for CO-BSI by comparing to a “gold standard” 48-hour definition.

**Findings:**

Among the population-based cohort of 14,106 episodes of BSI studied, 10,543 were classified as CO based on “gold standard” 48-hour criteria. When 2-day and 3-day definitions were applied, 10,396 and 10,707 CO-BSI episodes were ascertained, respectively. All but 147 (1.4%) true CO-BSI cases were included by using the 2-day definition. When the 3-day definition was applied, all cases of CO-BSI were identified but and additional 164 (1.5%) cases of hospital-onset HO-BSI were also included. Thus the sensitivity and specificity of the 2-day definition was 98.6% and 100% and for the 3-day definition was 100% and 98.5%, respectively. Overall, only 311 (2.2%) cases were potentially miss-classifiable using either the 2- or 3-calendar day based definitions.

**Conclusions:**

Use of either a 2- or 3-day definition is highly accurate for classifying CO-BSI.

## Introduction

Community onset (CO) bloodstream infections (BSI) are those that occur in outpatients or among those with first positive blood culture drawn less than 48 hours following admission to hospital [1]. These may be further categorized as healthcare-associated or community-acquired CO-BSI, and are mutually exclusive of hospital-onset (HO) BSI that are first identified 48 hours or more following hospital admission [1-3]. While dates of admission and of culture draw date are typically routinely recorded, exact times of these occurrences may not be readily available. Use of a calendar day-based definition that utilizes the difference in dates of first positive culture draw and admission may be used to classify CO-BSI. However, because a 48-hour time difference between admission and culture draw may occur over a 2 or 3 calendar day period, it is not known whether use of such calendar day-based definitions accurately ascertains the presence of a CO-BSI. The objective of this study was to evaluate the validity of calendar day- versus exact time-based definitions for CO-BSI.

## Methods

The base study population consisted of all Calgary, Canada, area residents with incident BSI occurring during 2000–2009 as previously described [4,5]. True cases of CO-BSI were established by applying the “gold standard” or reference CO-BSI definition based on the exact times listed for each of culture draw and hospital admission registration. Two CO-BSI study cohorts were then developed by applying 2 and 3-calendar day definitions to the overall cohort. In these cases, only dates, and not times, of culture draw and admission were considered. The 2-day definition included those where the date of culture was before, on the same day, or one day after admission, and the 3-day definition included those where the culture draw was before, on the same day, or within the next two days following admission to hospital. This study was approved by the ethics review board at the University of Calgary.

## Findings

During the study, there were 14,233 incident episodes of BSI; 10,543 were classified as CO and 3,680 as HO. Among this cohort, 127 episodes classified as HO-BSI had first positive cultures less than 48 hours following admission to one of the study hospitals as a result of either a recent hospital discharge or transfer from another institution and were excluded from further analysis. Among the 10,543 CO-BSI, 8,730 were admitted and 1,813 were not associated with an admission to hospital. The overall cohort admitted to hospital included 12,283 BSI cases of which 8,730 were CO and 3,553 were HO.

**Figure 1:**
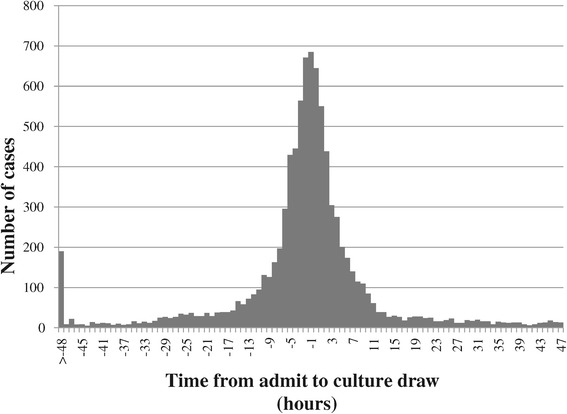
**Timing of blood culture draw following admission to hospital for community-onset bloodstream infections.**

Among the 8,730 true CO-BSI cases that were admitted to hospital, the median culture draw minus hospital admission time was −1 (IQR, −5 to 2) hours. The time of culture draw for these cases was distributed tightly around the time of admission as shown in Figure 1. Only 339 (3.9%) true CO-BSI cases were cultured between 24 and 47 hours following admission. In comparison, among the HO-BSI cases, the median time to culture draw was 12 (IQR, 6–24) days following admission. The distribution of these cases showed a progressive decrease from day 2. Of the HO-BSI cases, 290 (8%) were first cultured between 48–71 hours and 217 (6%) between 72 and 95 hours following hospital admission.

When the 2-day definition was applied, all but 147/10,543 (1.4%) true CO-BSI cases were included. When the 3-day definition was applied, all cases of CO-BSI were identified but and additional 164 (1.5%) cases of HO-BSI were also included. In other words, the sensitivity and specificity of the 2-day definition was 98.6% and 100% and for the 3-day definition was 100% and 98.5%, respectively. Overall, only 311 (2.2%) cases were potentially miss-classifiable using either the 2- or 3-calendar day based definitions. Among this cohort of 311 cases there were no evident features as regards gender, age, poly-microbial, antibiotic resistance, and species distribution that would allow their further classification as either CO- or HO-BSI.

## Discussion

In this study we show that use of either a 2 or 3 calendar-day based definition is highly accurate (>98%) to classify cases as to true CO-BSI cases based on a 48-hour definition. The ability to accurately classify CO-BSI arises due to the observation that the majority of CO cases are identified within a few hours of hospital admission (Figure 1). Use of the 2 calendar day definition results in the inclusion of only true cases, although it does fail to include a very small (1.4%) number of these cases. On the other hand, the 3-day definition results in the inclusion of all true CO-BSI cases albeit with the inclusion of a small number (1.5%) of misclassified HO-BSI cases. These data indicate that both the 2- or 3-day definitions are highly accurate for classification of CO-BSI with the former having slightly higher specificity and the latter sensitivity.

Accurate determination of CO-BSI is important for epidemiology and surveillance purposes. Traditionally BSI’s were classified based on attempts at defining location of acquisition infection with application of complicated definitions on a case-by-case review [6]. While standardized criteria are used in these cases, they still require a degree of subjective interpretation. As a result, reproducibility and inter-observer variation are considerable [7]. In contrast, CO-BSI are strictly defined by easily applied and objective time based criteria. These attributes allow objective inter-facility and regional comparisons and facilitate the use of electronic surveillance systems [8-10].

Strengths of this study include that we studied a large cohort of patients and that data were consistently and systematically recorded. Our system comprehensively identifies all patients with BSI in our population including those who are delayed in being admitted to hospital or who are not admitted to hospital for management of their BSI. We included all incident BSIs occurring in a well-defined population such that selection bias was minimized [4]. However, a potential limitation is that we defined the admission time as that registered within our hospital administrative database. This time is entered once an order has been written to admit the patient to hospital. The possibility exists that there could be delays from presentation/decision to admit the patient to hospital and the actual entry of the admission order as patients may have tests performed and assessments by consultants prior to establishing the patients admission disposition and order. However, this time is unlikely exceed a few hours at most and since the vast majority of CO-BSI occur with hours of admission, it is unlikely to have any significant effect on our study conclusions.

In summary, this study validates the practice of using calendar day based definitions for CO-BSI. Furthermore, these data indicate that 2- and 3-day based definitions are highly specific and sensitive and should be adequate for most surveillance purposes where exact times of admission and culture draw are not readily available.

## Abbreviations

BSI: Bloodstream infection
CO: Community-onset
HO: Hospital-onset
